# Relations of low contrast visual acuity, quality of life and multiple sclerosis functional composite: a cross-sectional analysis

**DOI:** 10.1186/1471-2377-14-31

**Published:** 2014-02-20

**Authors:** Johann Schinzel, Hanna Zimmermann, Friedemann Paul, Klemens Ruprecht, Katrin Hahn, Alexander U Brandt, Jan Dörr

**Affiliations:** 1 NeuroCure Clinical Research Center, Charité - Universitätsmedizin Berlin, Charitéplatz 1, 10117 Berlin, Germany; 2 Clinical and Experimental Multiple Sclerosis Research Center, Department of Neurology, Charité - Universitätsmedizin Berlin, Berlin, Germany; 3 Department of Neurology, Charité - Universitätsmedizin Berlin, Berlin, Germany

**Keywords:** Multiple sclerosis, Low contrast visual acuity, Sloan low contrast letter acuity, Multiple sclerosis functional composite, Quality of life, Retinal nerve fibre layer thickness

## Abstract

**Background:**

Although common and often disabling in multiple sclerosis (MS), visual dysfunction is currently not adequately accounted for in both clinical routine and MS trials. Sloan low contrast letter acuity (SLCLA) is a standardised chart-based measure of visual function particular at low contrast and has been suggested as additional visual component to the Multiple Sclerosis Functional Composite (MSFC). Here, we evaluate the relations between SLCLA, retinal integrity, MSFC, and quality of life (QoL) in MS patients.

**Methods:**

Cross-sectional analysis of retinal nerve fibre layer (RNFL) thickness, MSFC, SLCLA (2.5% and 1.25% contrast levels), visual evoked potentials, and QoL (Short Form (SF) 36, National Eye Institute Visual Functioning Questionnaire (NEIVFQ)) using baseline data of 92 MS patients from an ongoing prospective longitudinal trial. Relations between RNFL thickness or P100 latency and SLCLA were analysed using generalised estimating equations (GEE) accounting for intra-individual inter-eye dependencies and corrected for age, gender, and history of optic neuritis. Pearson’s correlations were used to assess relations between SLCLA, MSFC, and QoL.

**Results:**

SLCLA reflected RNFL thickness (p = 0.021) and P100 latency (p = 0.004) and predicted vision-related QoL, reflected by the NEIVFQ39 subscores “general vision” and “near activities” (p < 0.008 for both). SLCLA did not predict general QoL reflected by SF36. Implementing SLCLA into MSFC, thus creating a four-dimensional MSFC4, captured aspects of disability reflected by the NEIVFQ39 subscores “general vision” (r = 0.42, p < 0.0001) and “near activity” (r = 0.3, p = 0.014) which were not captured by standard MSFC3.

**Conclusions:**

SLCLA at 2.5% and 1.25% contrast levels correlates with retinal morphology and P100 latency and predicts some aspects of vision-related QoL in MS. More importantly, using a prospective cross-sectional approach we provide evidence that extending the MSFC by SLCLA as an additional visual component increases the performance of MSFC to capture MS-related disability. Longitudinal data on the relation between SLCLA, MSFC, and QoL will be available in the near future.

## Background

Afferent persistent visual dysfunction is frequent in multiple sclerosis (MS) [1] and is either due to incomplete recovery from optic neuritis (ON) or to rather diffuse damage of the optic pathway that may occur independent of ON [2-4]. According to a German study in which MS patients rated vision as the second most important physical function after ambulation, integrity of vision is of particular importance [5]. Likewise, several studies consistently showed that visual impairment compromises health related quality of life (QoL) [6,7]. Yet in clinical routine, the importance of visual function is not adequately accounted for, for a number of reasons: i) substantial vagueness exists concerning the morphological substrate of the visual deficit in patients without ON history; ii) bedside parameters for assessment of visual functions focus on high contrast (HC) visual acuity (VA) which is insensitive to change and to more diffuse and subtle visual dysfunction [8]; iii) potentially more sensitive measures like low contrast (LC) VA are rarely used in clinical routine; iv) patient-reported outcomes such as QoL that might disclose the impact of visual deficits on daily life are hardly addressed outside clinical trials; and v) most QoL questionnaires do not adequately capture vision-related aspects. Consequently, there is a need for better evaluation of more elaborate yet easy to perform measures of visual function and vision-related QoL assessment in MS patients.

**Figure 1:**
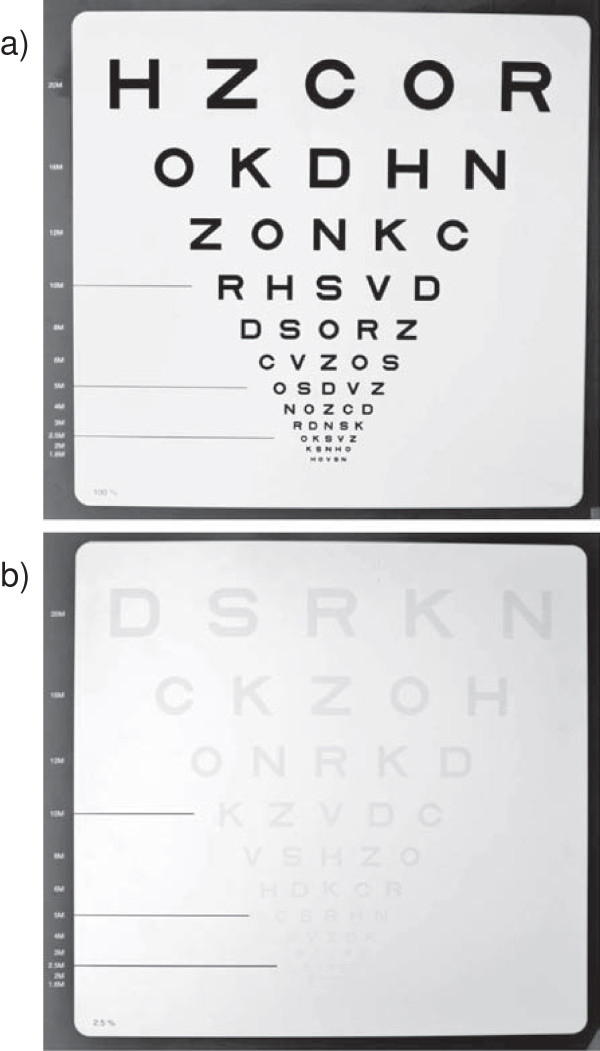
**Sloan low contrast letter acuity charts.** Depicted are Sloan low contrast letter acuity charts at 100% **(a)** and 2.5% **(b)** contrast level.

The Sloan low contrast letter acuity (SLCLA) charts comprise a set of letter charts based on the standardised format of the HC VA charts used in the early treatment of diabetic retinopathy study (ETDRS), but with different levels of contrast between letters (ranging from black to light grey) on white background (Figure 1). LCVA testing using SLCLA charts has been validated in MS patients and has been shown to capture unique aspects of neurologic dysfunction that were not captured by standard disability rating scales [9-11]. Moreover, SLCLA correlated with health-related QoL in MS patients [12].

Since its introduction in the late 1990s the Multiple Sclerosis Functional Composite (MSFC) has been established as major secondary outcome parameter for determination of disability in clinical MS trials [13]. Importantly, the MSFC accounts for the variability in health-related QoL measures not reflected by the expanded disability status scale (EDSS) [14]. Given the major shortcoming of the MSFC that it completely disregards vision as frequently impaired dimension in MS [8] considerations exist to complement the original MSFC with a fourth, visual component [8,15]; and SLCLA testing is considered a promising candidate [10,15]. However, formal prospective evaluation whether addition of SLCLA performance improves MSFC information overall has not yet been performed [15].

Using the baseline data set of an ongoing trial on the course of visual system parameters in MS patients we here evaluated the relation of SLCLA, morphological and functional visual system parameters, QoL, and MSFC in MS patients cross-sectionally. In particular we investigated whether implementation of SLCLA testing improves the performance of the MSFC.

## Methods

### Patients

We used the baseline data set of an ongoing, monocentric, longitudinal observational trial that evaluates changes of visual system parameters in MS patients over time (NCT01272596). Patients, aged 18 – 65 years, with a first episode of demyelinating CNS disease suggestive of MS (clinically isolated syndrome, CIS) and patients with definite MS according to the revised panel criteria [16] who had either relapsing remitting (RR) or secondary progressive (SP) disease course were eligible without restrictions regarding disease modifying treatments. Main exclusion criteria were MS relapses within the previous month, refractive errors > ± 8 dpt, and MS unrelated retinal pathologies, e.g. glaucoma or diabetic retinopathy. Of the 100 patients enrolled in the longitudinal trial, 92 patients were eligible for cross-sectional analysis. Eight patients had to be excluded because of exclusion criteria that became evident after enrolment or during the study (glaucoma, retinal ablation, severe myopia, unclear retinal pathology). A detailed cohort overview is given in Table 1.

**Table 1:** Cohort demographic and clinical data

**Subjects**	**n**	**92**
Gender (n/%)	Female	64/69.6
Age (years)	Mean ± SD	41 ±11
	Range	19-64
Disease duration (months)	Mean ± SD	96 ±70
	Range	2-295
Disease course (n/%)	RRMS	65/70.7
	CIS	21/22.8
	SPMS	6/6.5
EDSS	Median ± SD	2.0 ±1.4
	Range	0-7.0
Eyes with ON history (n/%)	No	122/66.3
	Yes	62/33.7
RNFLT (μm)	Mean ± SD	87.3 ±15.1
n = 184 eyes	Range	40.6 – 122.5
P100 VEP latency (ms)	Mean ± SD	115 ±16.7
n = 184 eyes	Range	75 - 177

*Abbreviations*: *EDSS* expanded disability status scale, *RNFLT* retinal nerve fibre layer thickness, *SD* standard deviation, *VEP* visual evoked potentials.

### Ethical statement

The study was approved by the local ethics committee and was conducted in accordance to the Declaration of Helsinki in its currently applicable version and the applicable German laws. All participants gave informed written consent.

### Low contrast visual acuity

LCVA was tested using SLCLA charts at four different contrast levels (100%, 10%, 2.5%, and 1.25%), meaning that the 100% level represents VA at HC. The 10% level was used for adaptation. SLCLA charts were standardised according to the ETDRS VA charts with five letters per line (Figure 1). Testing conditions were standardised with respect to illumination of the examination room (80-100 cd/m^2^), distance between patient and chart (2 m), position of patients (sitting), correction of visus (best available correction), and instructions given. The charts were scored based on the number of letters identified correctly (letter score, maximum score 60) [9]. All SLCLA tests were conducted binocularly (both eyes open) as this approach integrates possibly relevant binocular summation/inhibition effects [17], hence providing a measure of overall visual function closer to the “real life” situation than monocular testing. SLCLA per contrast level is given as number of letters identified correctly (max. 60).

### Multiple sclerosis functional composite

The conventional MSFC battery (MSFC3), comprising the 25 foot timed walk test (TWT), 9-hole peg test (9HPT) and paced auditory serial addition test (PASAT) was performed according to standardised procedures, calculated according to the standard manual, and normalised to this cohort [13]. A new “MSFC4” was created which additionally included SLCLA testing at 2.5% or 1.25% contrast levels. The MSFC4 score was calculated using SLCLA z-scores according to the formula (Z_9HPT_ + Z_T25FW_ + Z_PASAT_ + Z_Sloan_)/4, as previously described [10].

### Optical coherence tomography

Retinal nerve fibre layer (RNFL) thickness was determined using spectral domain optical coherence tomography (SD-OCT; Heidelberg Engineering, Germany; software version 5.1) according to previously reported scanning protocols [2]. Briefly, RNFL thickness around the optic disc was acquired using the 3.4 mm circle scan with the eye tracker system activated, and the maximum number of averaging frames in ART MEAN mode was tried to be achieved. All scans were performed by trained operators and were reviewed for scan quality according to the OSCAR IB consensus criteria [18]. Only eyes that passed the quality review were included in the analysis.

### Visual evoked potentials

Standard checkerboard stimulation was used for generation of visual evoked potentials (VEP). The electrodes were placed on Oz and Fz according to the “10-20 International System”. The analysed period was 500 ms following each visual stimulus; the P100 latency (ms) was recorded.

### Quality of life questionnaires

For assessment of QoL both the validated German versions of the self-administered questionnaires Short Form (SF) 36 and the National Eye Institute Visual Functioning Questionnaire (NEIVFQ) were used. SF36 was analysed according to normative data and scripts [19], providing the aggregate summary measures physical component summary (PCS) and mental component summary (MCS). The NEIVFQ, introduced in 1998 as a 51-item questionnaire, consists of 12 individually rated subscales and an overall composite (total) score [20]. Two additional versions comprising 39 and 25 items and a neuro-ophthalmic supplement are now available which are increasingly used in clinical MS research to address vision-related QoL aspects [1,12,21,22]. Here, we used the 39 item version [23] as at study initiation a validated German translation was available only for this version.

### Statistical analysis

Statistical analysis was performed using SPSS 21 (IBM, Somers, NY, USA). Distribution analysis of quantitative measures was performed using skewness, kurtosis and Kolmogorov-Smirnov tests. Pearson’s correlations were used to assess relations between MSFC, SLCLA, and QoL. Performance of MSFC4 was evaluated using partial correlations controlling for MSFC3. Relations between RNFL thickness or P100 latency and QoL were analysed using generalised estimating equations (GEE), a statistical model that accounts for intra-individual inter-eye dependencies. All GEE models were corrected for age, gender, and ON history. Statistical significance was established at p < 0.05. All analyses should be considered exploratory as no previous sample size calculation or adjustment for multiple testing was applied.

## Results

**Figure 2:**
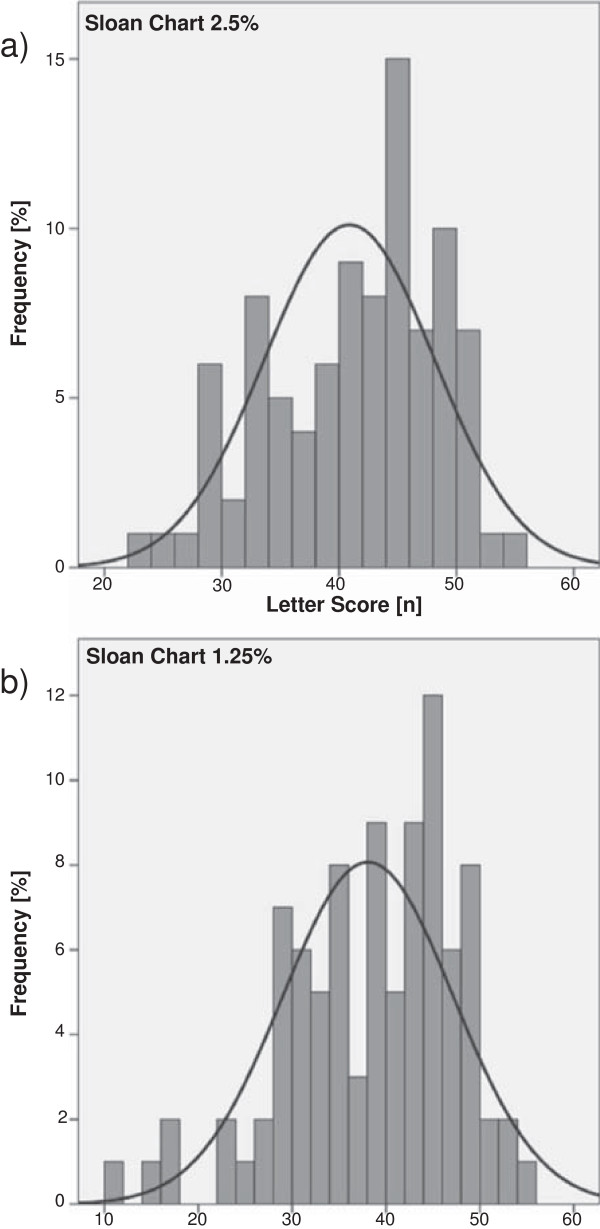
**Distribution analysis of Sloan low contrast letter acuity.** Depicted is the distribution of letter scores derived from Sloan low contrast letter acuity testing in the entire cohort at 2.5% **(a)** and the 1.25% **(b)** contrast level.

92 patients were included in the cross-sectional analysis. Basic demographic and clinical data are provided in Table 1, a summary of VA at both HC and LC levels and data on both general and vision-related QoL are given in Table 2. While as expected, VA at 100% and 10% levels showed strong ceiling effects, VA at 1.25% and particularly at 2.5% levels showed a near-normal distribution (Figure 2). Therefore, we focused on the 2.5% contrast level and provided additional data derived from 1.25% level where relevant.

**Table 2:** Visual acuity at different contrasts and quality of life data

			**Mean**	**Standard deviation**	**Maximum**	**Minimum**
Visual acuity (letter score)	Contrast levels	100%	58.5	3.0	60	42
		10%	53.5	5.5	60	36
		2.5%	41	7.5	55	23
		1.25%	38	9.0	54	11
SF36	PCS		44.48	9.72	63.72	19.17
	MCS		46.37	10.67	67.14	19.98
NEIVFQ	Total		88.08	8.02	99.44	58.11
	General health		59.5	18.7	100	15
	General vision		78.2	12.0	100	45
	Eye pain		80.5	17.6	100	25
	Near activity		87.3	12.6	100	41.7
	Distance activity		91.4	9.4	115	62.5
	Social functioning		96.2	7.3	100	66.7
	Mental health		89.5	12.5	118.8	35
	Role difficulties		78.5	17.9	100	31.3
	Dependency		96.5	13.7	100	12.5
	Driving		84.2	20.3	100	0
	Color		98	7	100	75
	Peripheral vision		88	15	100	50

*Abbreviations*: *SF36* Short Form 36 quality of life questionnaire, *NEIVFQ* National Eye Institute Visual Function Questionnaire, *PCS* physical component score, *MCS* mental component score.

### Correlations of low contrast visual acuity with retinal morphology and visual pathway function

To further characterise our cohort, RNFL thickness and P100 latency were determined (Table 1). Given recent studies showing close relations between retinal morphology and LCVA [24,25] we first investigated, whether SLCLA at the 2.5% level is predicted by RNFL thickness and, in a next step, by P100 latency. Using GEE analyses, which account for inter-eye relations in statistical models factoring ON history, age, and gender we observed a significant correlation between SLCLA at 2.5% and RNFL thickness (B = 4.562E-005; Standard Error (SE) = 1.983E-005; p = 0.021). Similar data were observed at the 1.25% level (B = 6.598E-005; SE = 2.468E-005; p = 0.008). Likewise, SLCLA at 2.5% (B = -4.013E-005; SE = 1.508E-005; p = 0.008) and SLCLA at 1.25% (B = -5.555E-005; SE = 1.920E-005; p = 0.004) were inversely correlated with P100 latency.

### Correlation of low contrast visual acuity with quality of life

We next investigated whether SLCLA reflects self-administered QoL assessment in MS patients. In contrast to previous reports [12], we did not observe a correlation between SLCLA at 2.5% and 1.25% levels and both the physical (PCS) and mental (MCS) component summary scores of the SF36 (Table 3). Given that SF36 is not designed to specifically capture vision-related QoL, we additionally used the vision-specific NEIVFQ39 which revealed a moderate correlation between SLCLA at the 2.5% (and also 1.25%) level and the NEIVFQ39 subscores “general vision” and “near activities” (Table 3). SLCLA was not associated with the NEIVFQ39 total score, which also includes subscores that seem rather irrelevant to MS patients like ocular pain or peripheral vision. Importantly, neither the total score nor any of the subscores was associated with VA determined at the 100% contrast level (not shown).

**Table 3:** Pearson correlation analyses of quality of life, multiple sclerosis functional composite, and Sloan low contrast letter acuity

			**TWT-z**	**9HPT-z**	**PASAT-z**	**MSFC3**	**SLCLA 2.5% z**	**SLCLA 1.25% z**	**MSFC4 (SLCLA 2.5%)**	**MSFC4 (SLCLA 1.25%)**
SF36	PCS	r	**0.369**	**0.324**	-0.004	**0.324**	0.197	0.177	**0.328**	**0.329**
		*p*	***0.001***	***0.004***	*0.97*	***0.004***	*0.078*	*0.114*	***0.003***	***0.003***
	MCS	r	0.093	-0.015	-0.103	-0.013	-0.032	-0.016	-0.021	-0.016
		*p*	*0.415*	*0.893*	*0.361*	*0.913*	*0.775*	*0.887*	*0.855*	*0.891*
NEIVFQ39	Total	r	0.043	**0.215**	0.055	0.145	0.178	0.123	0.182	0.164
		*p*	*0.693*	***0.044***	*0.608*	*0.177*	*0.093*	*0.248*	*0.092*	*0.129*
	General health	r	0.211	0.207	-0.007	0.197	0.082	0.072	0.191	0.194
		*p*	*0.05*	*0.053*	*0.95*	*0.065*	*0.441*	*0.5*	*0.076*	*0.072*
	General vision	r	0.021	0.159	-0.081	0.049	**0.279**	**0.274**	0.149	0.149
		*p*	*0.85*	*0.139*	*0.45*	*0.647*	**0.008**	**0.009**	*0.168*	*0.168*
	Eye pain	r	-0.09	-0.047	0.107	0.001	0.042	0.019	0.02	0.014
		*p*	*0.409*	*0.661*	*0.315*	*0.992*	*0.698*	*0.859*	*0.851*	*0.899*
	Near activities	r	0.177	**0.259**	0.046	**0.213**	**0.348**	**0.317**	**0.302**	**0.295**
		*p*	*0.101*	**0.015**	*0.664*	**0.046**	**0.001**	**0.002**	**0.004**	**0.006**
	Distant activities	r	-0.015	0.124	-0.13	-0.014	0.105	0.084	0.027	0.019
		*p*	*0.89*	*0.249*	*0.222*	*0.899*	*0.324*	*0.429*	*0.802*	*0.859*
	Social functioning	r	-0.021	0.116	-0.087	-0.002	0.122	0.092	0.042	0.031
		*p*	*0.846*	*0.28*	*0.413*	*0.988*	*0.252*	*0.387*	*0.696*	*0.774*
	Mental health	r	-0.031	0.158	-0.039	0.049	-0.082	-0.125	0.009	-0.007
		*p*	*0.779*	*0.143*	*0.713*	*0.653*	*0.443*	*0.242*	*0.931*	*0.946*
	Role difficulties	r	0.091	**0.239**	0.199	**0.264**	0.032	-0.014	**0.229**	**0.219**
		*p*	*0.402*	**0.025**	*0.06*	**0.013**	*0.766*	*0.893*	**0.033**	**0.042**
	Dependen-cy	r	0.043	0.129	-0.021	0.075	-0.048	-0.074	0.042	0.034
		*p*	*0.692*	*0.23*	*0.846*	*0.486*	*0.653*	*0.489*	*0.697*	*0.757*
	Driving	r	**0.289**	0.175	0.078	0.128	0.229	0.2	0.185	0.169
		*p*	**0.015**	*0.144*	*0.513*	*0.289*	*0.053*	*0.092*	*0.125*	*0.163*
	Color	r	0.019	0.066	0.006	0.048	0.062	0.022	0.065	0.05
		*p*	*0.865*	*0.55*	*0.957*	*0.665*	*0.569*	*0.842*	*0.557*	*0.654*
	Peripheral vision	r	0.142	0.095	0.134	0.172	0.151	0.099	0.203	0.187
		*p*	*0.199*	*0.391*	*0.217*	*0.117*	*0.165*	*0.366*	*0.065*	*0.09*

*Abbreviations*: *SF36* Short Form 36 quality of life questionnaire, *NEIVFQ* National Eye Institute Visual Function Questionnaire, *TWT* timed walk test, *9-HPT* 9-hole peg test, *PASAT* paced auditory serial addition test (3 sec. version), *SLCLA* Sloan low contrast letter acuity, *MSFC3* three-dimensional (conventional) multiple sclerosis functional composite, *MSFC4* four-dimensional multiple sclerosis functional composite including Sloan low contrast letter acuity, *r* Pearson correlation coefficient, *p* P-value, *PCS* physical health composite score, *MCS* mental health composite score.

Data depicted in bold reflect statistically significant data (p < 0.05).

### Implementation of low contrast visual acuity into MSFC

A key question of our still ongoing longitudinal study is whether the overall performance of MSFC would be improved by implementation of LCVA. Here, in this cross-sectional analysis of the baseline data of the longitudinal trial we thus asked whether SLCLA at 2.5% level improves the predictive value of MSFC with respect to QoL. Therefore, we first assessed the correlation of standard MSFC3 with QoL as determined by SF36 (Table 3). We found that the correlation of MSFC3 with the SF36 PCS (r = 0.324, p = 0.004) was mainly driven by TWT, whereas no correlation was observed between MSFC3 and the MCS. When implementing SLCLA (2.5% and 1.25%) into a four-dimensional MSFC4 we observed that MSFC3 and MSFC4 performed similar well (for MSFC4: r = 0.328, p = 0.003). This association was, however, completely lost in partial correlation analyses controlling for MSFC3, indicating that the correlation for MSFC4 and SLCLA can be completely explained by disabilities captured already by MSFC3.

Given the above described limitations of SF36 with respect to visual aspects we then used the NEIVFQ39 as a more vision-specific dependent variable. Both, MSFC3 and MSFC4 showed no correlation with total NEIVFQ39 scores but mild correlations with the subscores “near activities” and “role”. As these associations were mainly driven by the 9-HPT component in MSFC3 and by SLCLA and/or 9-HPT in MSFC4 (Table 3) we additionally performed partial correlations for MSFC4 and NEIVFQ39, controlling for MSFC3. Using this model revealed significant associations for the NEIVFQ39 subscores “general vision” (r = 0.42, p < 0.0001) and “near activities” (r = 0.3; p = 0.014). Similar results were found at the 1.25% level (“general vision”: r = 0.41; p = 0.001; “near activities”: r = 0.27; p = 0.024), suggesting that MSFC4 captures some aspects of vision-related QoL not covered by conventional MSFC3.

## Discussion

Using the cross-sectional baseline data set of an ongoing longitudinal trial on visual parameters in MS/CIS patients we here addressed the relations between SLCLA, retinal integrity, VEP, health-related QoL, and disability. Our main findings are i) SLCLA determined at 2.5% contrast level is predicted by RNFL thickness and P100 latency; ii) QoL determined by SF36 PCS reflects predominantly ambulation and, to a lesser degree, upper extremity function but not visual function as assessed by SLCLA; iii) in contrast, visual dysfunction in MS patients, determined by SLCLA, is captured by the “general vision” and “near activities” subscores of the NEIVFQ39; and iv) implementation of SLCLA as additional component might enhance the performance of the MSFC as it seems to capture visual dysfunction as QoL-relevant aspect of disability not covered by the conventional MSFC.

In line with recent studies on retinal morphology in relation to visual disability in MS [25,26] our data showing a significant association between SLCLA and retinal axonal integrity independent of a previous ON support the hypothesis that SLCLA reflects diffuse neurodegenerative processes in the retina, which could either be due to primary degeneration, secondary to subclinical inflammation, or both. In addition to morphological aspects, SLCLA testing also seems to capture physiologic function of the visual system as indicated by the significant association with P100 latency. The ability of SLCLA testing to capture visual deficits that have a major impact on the QoL of MS patients but are not adequately captured by standard visual assessments has been addressed in another study by the same group [12]. When using the 25-item version of the NEIVFQ plus the 10-item neuro-ophthalmic supplement in a cohort of 167 patients with RR or progressive MS, the authors observed significant correlations between both HCVA and LCVA with both total score and most of the subscores, with “near activities” “role difficulties” and “driving” subscores showing the strongest correlations. Notably, associations between SLCLA and QoL were reported to remain significant when controlling for HCVA. Interestingly, that study also revealed significant correlations between SLCLA and the PCS of SF36 in a sub-cohort of 115 patients. In contrast, in our cohort, LCVA was reflected only by the subscores “general vision” and “near activities”, while HCVA was not related to QoL. Moreover, in our study, SLCLA did not predict QoL as determined by SF36, a difference that cannot be explained by different sample sizes. Our observation that only particular NEIVFQ subscores were correlated with SLCLA raises the question which aspects of visual demands in daily life are covered by the respective subscores. “General vision” can be considered a more common aspect that is important in daily life. Thus, already mild reduction of LCVA might easily interfere with the “general vision” aspect. The item “near activities” reflects all actions at near distance and is thus affected by tasks requiring accurate execution or ability to distinguish small or pale objects, e.g. reading, writing, or painting. Thus, correlation of this aspect with impairment of LCVA seems to be likely. In contrast, as ocular pain for example is not a typical feature in MS outside acute ON one would not expect interference with the “eye pain” subscore of the NEIVFQ. While in the study by Mowry et al. [12] a different measure of visual performance, a different version of NEIVFQ, and a larger sample size was used, which impedes a direct comparison with our data and may account for some of the observed discrepancies, the bottom line information of both studies are that in MS reduction of SLCLA negatively impacts QoL and that the NEIVFQ is a promising tool to capture vision-related QoL. Our data strengthen the roles of LCVA testing and NEIVFQ for evaluation of visual disability and its impact on QoL in MS.

Against this background SLCLA testing is a promising candidate to complement the current three-dimensional MSFC3 by a fourth visual component in order to compensate for its obvious limitation that it does not cover visual dysfunction. Earlier retrospective studies already suggested that such a four-dimensional MSFC4 would indeed capture relevant visual aspects of disability not covered by EDSS or standard MSFC [10]. A recent review on disability outcome parameters in MS deemed formal inclusion of SLCLA test in the MSFC appropriate, but demanded prospective testing to confirm whether such an extension improves MSFC performance overall [15]. Here, we evaluated the inclusion of SLCLA in the MSFC in a prospective approach. QoL was chosen as dependent variable as this parameter is increasingly considered important when evaluating disease progression and treatment effects in MS. Despite the availability of more MS-specific QoL tools we opted for the SF36 as this tools has been used for the initial validation of the MSFC and in numerous other trials [12,14]. In keeping with the observation of Heesen at al. that in the self-concept of MS patients ambulation has the highest significance [5] the correlation between standard MSFC and the PCS of the SF36 in our cohort was mainly driven by TWT. Although vision was the second important function in the study by Heesen [5], in our cohort this observation was not reflected by SF36-determined QoL when SLCLA was included in a model controlling for MSFC3. The most likely explanation and the rationale for thereafter using the NEIVFQ is that SF36 is too insensitive a measure with respect to vision. Using NEIVFQ in the same model controlling for functions captured by standard MSFC3 revealed that the inclusion of SLCLA testing at the 2.5% (and 1.25%) level captured additional aspects of disability that affect at least some aspects of vision-related QoL, reflected by the “general vision” and “near activities” subscores. As a conclusion, these cross-sectional findings may cautiously suggest implementation of SLCLA testing in the MSFC as an option to increase the ability of the MSFC to capture MS-relevant disability.

Several limitations of our study need to be addressed. First, data are based on cross-sectional analyses of the baseline data set of an ongoing clinical trial. Whether MSFC4 is also superior to MSFC3 with respect to longitudinal intra-individual changes of LCVA remains to be evaluated; longitudinal data are underway. Second, the majority of included patients had either CIS or RRMS with a median EDSS of 2.0. Thus, our cohort represents rather mildly disabled MS patients, and more severely disabled patients or patients with progressive disease are certainly under-represented. Third, we observed a few outliers with rather unaffected SLCLA but low NEIVFQ39-scores. Having a closer look at these particular patients, mainly the NEIVFQ39 subscores “role”, “peripheral vision”, “mental health”, “eye pain” and “dependency” were the worst rated. Although these aspects are probably not closely related to MS-specific aspects of visual disability we did not find any other relevant ocular disease in these patients. Hence, we refrained from excluding these patients from the analyses.

## Conclusions

In accordance with previous data, SLCLA reflects both retinal morphology and (patho)physiologic function of the visual system and predicts vision-related QoL in MS as determined by NEIVFQ39. More importantly, we here showed in a prospective cross-sectional approach that SLCLA is a promising tool for extending the MSFC by a fourth visual component. Longitudinal data on the relation between SLCLA tests, MSFC and QoL will be available in 2014.

## Competing interests

This work was supported by a limited research grant by Novartis Pharma to JD. The author(s) declare that they have no further competing interests.

## Authors’ contributions

JS evaluated the patients, analysed data and drafted the manuscript. HZ evaluated the patients and made substantial contributions to analysis and interpretation of data. FP made substantial contributions to conception and design of the study and interpretation of data. KR and KH made substantial contributions to acquisition and interpretation of data. AB supervised the analysis and interpretation of data. JD was in charge of study conception and design, supervised acquisition and interpretation of data and drafted the manuscript. All authors critically revised the manuscript for important intellectual content. All authors read and approved the final manuscript.

## Pre-publication history

The pre-publication history for this paper can be accessed here:

http://www.biomedcentral.com/1471-2377/14/31/prepub
